# Experience of source isolation during hospitalization – a qualitative study

**DOI:** 10.1186/2047-2994-4-S1-P95

**Published:** 2015-06-16

**Authors:** AF Madsen

**Affiliations:** 1 Department of Clinical Microbiology, Rigshospitalet, Copenhagen, Denmark

## Introduction

This study explored and describes the factors that may influence how patients react to source isolation from others during hospitalization.

## Objectives

The study also sought to determine how background variables such as gender, age and previous hospitalization affect source isolation.

## Methods

This qualitative study used content analysis to review data collected from interviews with five patients.

- The conceptual framework describes antibiotic resistance and infection control from a public health perspective and explored its prevention in Denmark.

- The theoretical framework describes how patients experience an infection acquired by exposure to drug-resistant bacteria, as well as subsequent isolation.

## Results

The limited space, lack of contact with people resulted in patient monotony and anxiety.

- Women showed greater concern about precautions against infection, and about risk of transmitting disease to visitors.

- Men outwardly resigned themselves to the situation and did not speculate about infection precautions. Men had a more rational approach, and tended to cope better when isolated.

- Younger patients seemed to have better coping strategy during isolation.

- Elderly patients felt sad and lonely.

- Patients developed their own strategies for coping with source isolation.

## Conclusion

Hospitals need more alternatives (e.g., better training and improved treatment) to prevent negative psychological affects due to isolation without compromising infection prevention. Hospitals should update their personnel at all organization levels, and focus on room facilities in the ward, contact time and improved information. Risk assessment should be individualized for each patient.

## Disclosure of interest

None declared.

